# A Review of Research on Disparities in the Care of Black and White Patients With Cancer in Detroit

**DOI:** 10.3389/fonc.2021.690390

**Published:** 2021-07-07

**Authors:** Michael S. Simon, Sreejata Raychaudhuri, Lauren M. Hamel, Louis A. Penner, Kendra L. Schwartz, Felicity W. K. Harper, Hayley S. Thompson, Jason C. Booza, Michele Cote, Ann G. Schwartz, Susan Eggly

**Affiliations:** ^1^ Department of Oncology, Wayne State University, Detroit, MI, United States; ^2^ Population Studies and Disparities Research Program, Karmanos Cancer Institute, Detroit, MI, United States; ^3^ Department of Hematology/Oncology, Ascension Providence Hospital/Michigan State University College of Human Medicine (MSUCHM), Southfield, MI, United States; ^4^ Department of Family Medicine and Public Health Sciences, Wayne State University, Detroit, MI, United States; ^5^ Department of Academic and Student Programs, Wayne State University, Detroit, MI, United States

**Keywords:** disparities, co-morbidities, cancer treatment, physician-patient communication, socio-economic

## Abstract

Racial disparities in cancer incidence and outcomes are well-documented in the US, with Black people having higher incidence rates and worse outcomes than White people. In this review, we present a summary of almost 30 years of research conducted by investigators at the Karmanos Cancer Institute’s (KCI’s) Population Studies and Disparities Research (PSDR) Program focusing on Black-White disparities in cancer incidence, care, and outcomes. The studies in the review focus on individuals diagnosed with cancer from the Detroit Metropolitan area, but also includes individuals included in national databases. Using an organizational framework of three generations of studies on racial disparities, this review describes racial disparities by primary cancer site, disparities associated with the presence or absence of comorbid medical conditions, disparities in treatment, and disparities in physician-patient communication, all of which contribute to poorer outcomes for Black cancer patients. While socio-demographic and clinical differences account for some of the noted disparities, further work is needed to unravel the influence of systemic effects of racism against Black people, which is argued to be the major contributor to disparate outcomes between Black and White patients with cancer. This review highlights evidence-based strategies that have the potential to help mitigate disparities, improve care for vulnerable populations, and build an equitable healthcare system. Lessons learned can also inform a more equitable response to other health conditions and crises.

## Highlights

An understanding of race-related factors underlying and maintaining health disparate outcomes is essential for developing interventions and initiatives that could reduce current inequalities in cancer care. Knowledge gained from research on racial health disparities can also help to eradicate disparities in the future.

## Introduction

In the United States, there are significant racial disparities in cancer incidence and outcomes with higher incidence rates and worse outcomes occurring in Black compared to White populations across multiple primary cancer sites (1). Evidence suggests that these disparities result in large measure from inequitable social, economic, political, behavioral, and psychological processes, which disproportionately negatively impact outcomes among Black individuals with cancer (2). Numerous studies have evaluated the extent to which levels of socioeconomic status (SES), access to care, cancer treatment, and clinical communication contribute to racial health and healthcare disparities (3). While multiple causal pathways might explain in part why Black cancer patients have worse outcomes than their White counterparts, the major underlying force is arguably the legacy of various forms of racism against Black people in the US (4).

Systemic racism in the US began with the legal enslavement primarily of people from Africa and has pervaded medical practice over the last 300 years. Examples of racism in the US medical system range from unethical experimentation on coerced Black people to institutional practices in medical care that either exclude Black people entirely or systematically provide them with poorer treatment than White people (5–10). In an effort to understand and address Black-White health disparities, researchers in the Karmanos Cancer Institute’s (KCI’s) Population Studies and Disparities Research (PSDR) Program have conducted research over the past 30 years ranging from descriptive to evaluative and interventional. Our efforts to explore and better understand racial disparities in cancer incidence, care, and outcomes, along with efforts of our collaborators, are particularly relevant to our institution given our location in Detroit Michigan, a city with a majority of Black people.

In 2019, it was estimated that 69.6% of Michigan’s 1,350,329 non-Hispanic Black population resided within the tri-county Detroit area with the largest proportion of the population (N=518,305) living in the city of Detroit, and the remainder (N=421,799) living in the suburban environs surrounding Detroit. According to Surveillance Epidemiology and End Results (SEER) data, between 2013 and 2017, Black compared with White individuals in Detroit had a disproportionate share of the burden of cancer, with incidence and death rates per 100,000 of 491.92 *vs* 489.01 and 195.25 *vs* 164.68 respectively (11). Our research on disparities has largely focused on populations in Detroit and the surrounding tri-county area. Further, given that KCI is one of the founding sites of the National Cancer Institute’s SEER program, many of our studies and those of our collaborators include populations outside of the Detroit area.

In this review, we present a selection of research studies published on cancer disparities conducted by investigators in the KCI’s PSDR program together with collaborators from several different institutions. After a thorough PubMed search of publications by PSDR investigators on racial disparities, we selected studies which focused on Black-White disparities in clinical presentation at diagnosis, treatment, and outcomes, as well as those that evaluated interventions designed to reduce or eliminate Black-White disparities. Given the critical juncture in race relations and health care equity in the US, the goal of this review is to summarize and critique published research and to provide a framework to shape future research that can lead to elimination of health disparities.

## Generations of Disparities Research

The sections of the review generally follow the framework of Thomas et al. (12), which describes three generations of cancer disparities research ranging from descriptive, to analytical and interventional. First generation studies are those that both identify and document the existence of health disparities. Second-generation studies are analytic or evaluative, and attempt to assess variables that could potentially explain the noted disparities. Lastly, third generation studies have the goal of testing interventions that could serve as solutions to mitigate disparate outcomes. While the order of this review generally follows this overall framework, many of the studies cited span both first and second generations, and the section focused on physician-patient communication includes studies spanning all three generations of disparities research. We will conclude with a discussion of fourth generation research, the goal of which is to ultimately take action to eliminate disparities.

## First-Generation Studies: Documenting Cancer Disparities

### Black-White Disparities in Phenotypic Features at Diagnosis

**Table 1** lists studies by PSDR investigators and their collaborators that identify and document disparities stratified by primary cancer type and show first-generation evidence that Black patients as compared with White patients present with more advanced and aggressive disease at diagnosis across cancer sites evaluated. In a survival analysis of 10,502 women with breast cancer using data from the Detroit Metropolitan Area (DMA)-SEER database, Black women were more likely to present with regional or distant stage disease (44.5%) compared to White women (36.5%) (p <0.00001) (13). In another analysis of 1,700 women with early-stage breast cancer, using data from the Detroit and Los Angeles SEER registries, 16.1% of patients with stage 0 + 1 disease were Black and 75% were White, compared to stage II + III disease where 21.2% were Black and 62.4% were White (14). Similar results were reported for other primary cancer sites including a Detroit Metropolitan Area–SEER analysis of colorectal cancer (CRC) (15) and a study of young-onset CRC identified at 18-SEER sites, which showed that Black compared to White patients were less likely to be diagnosed with early-stage disease (16).

**Table 1:** First-Generation Evidence of Black-White Disparities at Diagnosis.

Years of Study, Reference	Study Population	Clinical Presentation	Black Patients (%)	White Patients (%)	P-value
**Breast Cancer**					
12/2002- 1/2013, Lantz et al. (14)	DMA^2^ + Los Angeles –SEER^3^ N=1,700	Stage (AJCC, TNM)^1^			<0.001 (row percentage)
		0 + I	16.1	75	
		II + III	21.2	62.4	
1988-1992, Simon and Severson (13)	DMA SEER N=10,502	Stage (SEER)			<0.00001
		Local	53.6	62.7	
		Regional	38.2	33.1	
		Remote	6.3	3.4	
		Unknown	1.9	0.8	
		Tumor Size			<0.00001
		T1	46.5	62.3	
		T2	41.2	31.7	
		T3	11.0	5.3	
		T4	1.3	0.7	
		Histologic Grade			<0.00001
		1	3.2	4.3	
		2	15.3	14.4	
		3	27.1	16.6	
		4	2.7	1.5	
		Unknown	51.7	63.2	
1996-2005, Roseland et al. (17)	HFHS^4^ N=2,387	Tumor Size			<0.001
		< 2 cm	56	66	
		2.1-5 cm	32	28	
		> 5 cm	9	4	
		Lymph Nodes			0.002
		Negative	66	72	
		Positive	34	28	
		Grade			< 0.001
		Well/moderate	51	63	
		Poor/undifferentiated	45	32	
		Hormone Receptor			<0.001
		ER/PR: Positive	64	75	
		ER/PR: Negative	30	19	
1994-1997, Du and Simon (18)	KCI^5^ N=588	Stage (TNM)			0.001
		I	42	51	
		II	47	46	
		IIIA	5	1	
		IIIB	6	2	
		Lymph Nodes +	39	36	0.563
		Hormone Receptor			<0.001
		ER+	52	73	
		PR+	49	65	
Holowatyj et al. (24)	SEER-18 N=134,639	Hormone Receptor			<0.0001
		ER+, PR +	8.2	73.2	
		ER+/PR-	12.9	68.7	
		ER-/PR+	19.9	59.7	
Holowatyj et al. (28)	DMA-SEER N=2,216	21-Gene Recurrence Score			0.0004
		< 18	55.9	60.8	
		18-30	29.3	30.9	
		≥31	14.8	8.3	
**Colorectal Cancer (CRC)**					
1988-1992, Yan et al. (15).	DMA-SEER N=9,078	Stage (TNM)			<0.001
		I + II	37.2	45.1	
		III	19.9	21.2	
		IV	27.6	22	
2000-2009, Holowatyj et al. (16).	SEER-18 N=28,145	Stage (ACJCC)			<0.001
		0	2.1	2.6	
		I + II	36.1	40.2	
		III	28.7	29.9	
		IV	27.1	21.7	
		Grade			<0.001
		I	7.1	8.2	
		II	62.5	59.8	
		III	16.2	18.5	
		IV	1.1	1.3	
**GYN Cancers**					
2000-2013, Park et al. (30).	SEER -18 N=76,241 (ovarian)	Stage (SEER)			
		Local	12.5	15.9	
		Regional	8.5	8.7	
		Distant	68.7	67.6	
		Un-staged	10.4	7.8	
		Grade			
		Low	14.5	17.6	
		High	36.4	45.6	
		Unknown	49.1	36.9	
1988-1992 Movva et al. (48)	DMA-SEER N=1,036 (cervical)	Stage (FIGO^6^)			<0.001
		I	48.9	59.6	
		II	19.6	18.2	
		III	12.7	8.8	
		IV	8.5	7.1	
2000-2011,Cote et al. (23)	SEER-18: N=120,513 (uterine)	Stage (SEER)			
		Local	53	70	
		Regional	24	18	
		Distant	16	8	
		Unknown	4	4	
		Grade			
		Low	43	65	
		High	36	21	
		Unknown	20	14	
**Prostate Cancer**					
1988-1992, Schwartz et al. (27)	DMA-SEER N=8,679	Grade (Localized)			<0.001
		Well	31	30	
		Moderate	43	47	
		Poor/Undifferentiated	18	14	
		Missing	8	9	
		Grade (Regional)			0.21
		Well	10	7	
		Moderate	44	49	
		Poor/Undifferentiated	37	37	
		Missing	10	7	
7/1990-12/1999 Powell et al. (53)	KCC N=791	Clinical Stage			0.11
		T1a-T1b	3.49	2.15	
		T1c	27.07	33.67	
		T2a	43.17	25.26	
		T2b	17.9	12.88	
		T2c	7.42	6.26	
		T3	0.44	0.18	
1992-2001 Powell et al. (25)	DMA-SEER N=1,056	Mean Tumor Volume (cc) by age group			
		40-49	0.436	0.215	
		50-59	0.941	0.899	
		60-69	0.875	2.555	
		70-79	0.562	2.941	
		% Gleason Score (≤ 6) by age group			
		40-49	97	100	
		50-59	87	93	
		60-69	86	87	
		70-79	65	84	
1973-1994 (pre-PSA) and 1995-2005 (PSA) Powell et al. (26)	SEER-18 N=Pre PSA 212,719 Post PSA 309,793	% Gleason Score (by age group)			P<0.0001
		**40-49 years**			
		2-6	45.4	52.4	
		7-10	54.6	47.6	
		**50-59 years**			
		2-6-	37.6	44.8	
		7-10	62.4	55.2	
		**60-69 years**			
		2-6	32.4	37.4	
		7-10	67.6	62.6	
**Other Primary Sites**					
2002-2007 Schwartz et al. (34)	DMA-SEER: N=951 Renal Cell Carcinoma	Stage (AJCC)			0.03
		I	72	65	
		II	12	11	
		III or IV	13	20	

^1^AJCC, TNM: American Joint Committee on Cancer, Tumor, Nodal, Metastases.

^2^DMA, Detroit Metropolitan Area.

^3^SEER, Surveillance, Epidemiology and End Results.

^4^HFHS, Henry Ford Health Systems.

^5^KCC, Karmanos Cancer Center.

^6^FIGO, Federation of Gynecology and Obstetrics Staging System.

Data from single institutions in the Detroit Metropolitan area also showed disparities in stage at diagnosis. Together with their collaborators, researchers from the PSDR conducted studies using data from Henry Ford Health System (HFHS), a large integrated health center in Detroit, and the Karmanos Cancer Center (KCC), one of 54 National Cancer Institute designated Comprehensive Cancer Centers in the US.

In a study of Black-White differences in breast cancer survival among women diagnosed and treated at HFHS, the distribution of tumor size by race demonstrated that 66% of White women had tumors ≤ 2cm and 4% had tumors > 5 cm; however, the tumor size distribution for Black women was 56% and 9% respectively (p< 0.001) (17). Using data from the KCI, PSDR investigators demonstrated that Black women were also more likely to present with advanced disease at diagnosis (18).

Other first-generation evidence includes studies that describe Black-White disparities in tumor phenotypic characteristics in both SEER-based and single institution studies. Black compared to White cancer patients across multiple tumor types were more likely to present with higher-grade, and more aggressive disease (16, 19–22), and among individuals with endometrial cancer, Black patients were more likely to present with histologic subtypes associated with worse outcomes (20–23). In three hospital-based studies, Black women were more likely to present with triple-negative breast cancer (17, 18, 24), and in studies of prostate cancer, Black men were more likely to present with aggressive disease associated with higher Gleason grade and greater prostate gland volume (25–27). Similar patterns were seen in a study evaluating tumor genomic profiling in a Detroit Metropolitan Area-SEER analysis of women with early-stage hormone-sensitive breast cancer. The results from this analysis demonstrated higher recurrence scores in Black compared to White women, signifying a greater need for adjuvant chemotherapy (28).

These racial disparities in stage and tumor phenotype have been exemplified in reported racial disparities in overall survival over time. In an analysis of 25,997 women with breast cancer diagnosed through the Detroit Metropolitan Area–SEER registry between 1975-2001, successive historical cohorts (1975-1980 and 1990-1995) demonstrated a widening survival gap between Black and White women with breast cancer over time. This disparity pertained specifically to younger women who were not yet Medicare-eligible. In addition, disadvantages in access to radiation, chemotherapy, and hormonal therapy continued over time, particularly among Black women with lymph node-positive disease (data not shown in Table) (29). This information serves in a sense as a “natural experiment” in which it can be hypothesized whether changes in policy and interventions over time could have an influence on cancer survival and treatment. Lastly, in a SEER-18 study of ovarian cancer, Black compared to White women experienced poorer 5-year overall survival for each stage of disease (30).

### Black-White Disparities in Co-Morbid Medical Conditions Among Patients With Cancer

In this section, we include studies of racial disparities in comorbidities among patients with cancer in order to identify factors that may place Black cancer patients at greater risk for poorer outcomes. In studies using data from both single institutions to multiple sites, PSDR investigators and collaborators found that Black compared to White cancer patients across multiple tumor types were more likely to be diagnosed with co-morbid medical conditions including hypertension (HTN) (18, 21, 22, 31, 32), diabetes (18, 32), heart disease (18), obesity (22, 33, 34), and chronic renal failure (34).

**Table 2** lists studies suggesting that co-morbid medical conditions have a differential impact on cancer outcomes for Black and White patients with cancer. In a case-control study from the HFHS, metabolic syndrome was associated with prostate cancer risk in Black men with organ confined disease but not in White men (OR 1.82, 95% CI 1.02-3.23). Data from this study also suggested a possible protective influence of obesity for White but not Black men (OR 0.51, 95% CI 0.33-0.8) (32). Another analysis of Black ovarian cancer survivors from a large multi-site epidemiological study, showed that BMI ≥ 40 and weight gain since age 18 were associated with higher odds of ovarian cancer (OR 1.72, 95% CI 1.12-2.66 for BMI; and OR 1.52, 95% CI 1.07-2.16 for weight gain) (35).

**Table 2:** The Impact of Co-Morbid Medical Conditions.

Years of study, Reference	Population	Outcome	Race Groupings	HR, 95% CI	Disparity
1999-2004, Beebe-Dimmer et al. (32)	HFHS^1^ N=637 cases, 244 controls	Association between metabolic syndrome (MetS) and prostate cancer	MetS and prostate cancer:	Odds Ratio:	Metabolic syndrome associated with prostate cancer risk in Black men with organ confined disease. Obesity protective for White and not Black men.
			Black	1.71 (0.97-3.01)	
			White	1.02 (0.64-1.62)	
			Organ confined with MetS:		
			Black	1.82 (1.02-3.23)	
			White	1.01 (0.63-1.62)	
			Advanced with MetS		
			Black	0.93 (0.31-2.77)	
			White	1.17 (0.55-2.51)	
			Obesity		
			Black	1.15 (0.70-1.89)	
			White	0.51 (0.33-0.8)	
2010-2011, Bandera et al. (35)	AACES^2^ N=492 cases, 696 controls All Black participants	Impact of BMI^3^ 1yr pre-diagnosis and weight gain since age 18 on ovarian cancer risk	Ovarian cancer risk BMI ≥40	Odds Ratio	Ovarian cancer risk associated with higher BMI and weight gain in study of Black postmenopausal women.
				1.72 (1.12-2.66)	
				Ptrend 0.03	
			Weight gain since age 18	1.52 (1.07-2.16)	
				Ptrend 0.02	
2002-2007, Colt et al. (31)	USKCS^4^ Cases: 843 White and 358 Black; Controls: 707 White and 519 Black	Role of hypertension in renal cell cancer incidence by race	HTN risk:	Odds Ratio	Higher risk of renal cell carcinoma for Black *vs.* White patients with HTN and consistent risk with prolonged or poorly controlled HTN.
			Black	2.8 (2.1-3.8)	
			White	1.9 (1.5-2.4)	
			Risk after 25 years of HTN:		
			Black	4.1 (2.3-7.4)	
			White	2.6 (1.7-4.1)	
				Ptrend <0.001	
			Risk with poorly controlled HTN:		
			Black	4.5 (2.3-8.8)	
			White	2.1 (1.2-3.8)	
				Ptrend<0.001	
Callahan et al. (36)	USKCS N=965 cases, 953 controls KPNC^5^: N=2162 cases, 21,484 controls	Race and gender-specific PAR% for hypertension and CKD based on race, age ≥50 years	Hypertension (USKC):	PAR%^6^:	Black compared with White patients had larger population attributable risk percent associated with HTN and chronic kidney disease.
			Black male	44.4% (24.7-64.1%)	
			White male	26.6% (14.2-39%)	
			Black female	50% (23.5-76.7%)	
			White female	28.5% (13.4-43.64%)	
			Hypertension (KPNC):		
			Black male	22.8% (1.6-44.1%)	
			White male	18.9% (13.7-24.1%)	
			Black female	39.8% (17.5-62.2%)	
			White female	27.4% (20.3-34.5%)	
			CKD^7^ (USKC):		
			Black male	9.4% (4.0-14.8%)	
			White male	0.6% (-0.5-1.6%)	
			Black female	8.4% (1.9-14.9%)	
			White female	0.4% (-1.5-2.3%)	
			CKD (KPNC):		
			Black male	10.1% (4.6-15.5%)	
			White male	0.0% (-0.6-0.5%)	
			Black female	6.9% (1.5-12.4%)	
			White female	-0.3% (-0.8-0.1%)	
1986-1989 Silverman et al. (38)	SEER registries: Atlanta, Detroit, and New Jersey N=526 cases, 2,153 controls	Role of comorbidities in pancreatic cancer incidence by race	PAR %- smoking, diabetes, family history:	PAR%:	The known pancreatic risk factors accounts for some of the difference in risk for Black and White men, however a larger proportion of the difference in risk is accounted for by less known risk factors in Black and White women.
			Black male	46% (10-82%)	
			White male	37% (13-62%)	
			Black female	15% (13-43%)	
			White female	27% (4-49%)	
			PAR% adding heavy alcohol use, high BMI:		
			Black male	53% (13-93%)	
			White male	49% (23-74%)	
			Black female	88% (66-111%)	
			White female	47% (2-92%)	
2000-2005 Olson et al. (37)	SEER^8^ Medicare: N=11,610 White, and 958 Black; age ≥ 66 years	Influence of co-morbidities on overall survival in endometrial cancer	OS^9^:	Multivariate	Black-white differences in OS and DSS in multivariable analysis remained after adjusting for co-morbidities
			Black *vs.* White	1.16 (1.05-1.28)	
			DSS^10^		
			Black *vs.* White	1.27 (1.08-1.49)	
1990-2005, Ruterbusch et al. (22)	HFHS N=627	Influence of comorbid conditions on survival: endometrial cancer	Black *vs.* White:	Multivariate	No Black-White differences in overall survival, but continued differences in disease specific survival after adjusting for clinical factors and co-morbid conditions
			Death from any cause	1.22 (0.94-1.57)	
			Death from endometrial cancer	2.27 (1.39-3.68)	
1996-2012, Cote et al. (21)	KCC^11^: N=97 White & 89 Black	Post-surgical outcomes, survival in very obese women (BMI ≥40): endometrial cancer	Overall Survival	Multivariate	Black-White difference in post-op complications but no differences in overall or disease-specific survival after adjusting for age, histology, FIGO stage and grade, treatment and comorbidities.
			Black *vs.* White	0.85 (0.36-2.03)	
			Disease specific survival		
			Black *vs.* White	0.95 (0.26-3.52)	

•All statistics other than hazard ratios are indicated in the table.

^1^HFHS, Henry Ford Health System.

^2^AACES, African American Cancer Epidemiology Study.

^3^BMI, Body Mass Index.

^4^USKCC, US Kidney Cancer Study.

^5^KPNC, Kaiser Permanente Northern California.

^6^PAR%, Population attributable risk percent.

^7^CKD, Chronic kidney disease.

^8^SEER, Surveillance, Epidemiology and End Results Program.

^9^OS, Overall survival.

^10^DSS, Disease specific survival.

^11^KCC, Karmanos Cancer Center.

A study from the US Kidney Cancer Study (USKCS), which included Detroit as one of two sites, compared risk of renal cell carcinoma (RCC) among Black compared to White patients. Findings demonstrated a higher risk of RCC associated with history of HTN among Black compared to White patients (OR 2.8, 95% CI 2.1-3.8) (31). In an expansion of the USKCS analysis, which also used data from the Kaiser Permanente Northern California (KPNC) registry, the population-attributable risk percentages (PAR%) for HTN and RCC were highest among Black women followed by Black men, White women, and White men. The PAR% for RCC for chronic kidney disease for Black men and women was 7-10 times greater than for White women and men (36).

An analysis of the impact of co-morbid medical conditions on disparities in survival among Black and White women with endometrial cancer at the HFHS, found that Black women continued to have worse overall survival outcomes despite adjustment for co-morbid medical conditions (22). These findings were replicated in a similar analysis of SEER-Medicare linked data (37). In comparison, however, in a study of morbidly obese women with endometrial cancer from the KCC, there were no Black-White disparities in overall or disease specific survival, suggesting the possibility of more equal provision of care once women are part of a single medical care system (21).

Lastly, in a collaborative population-based case-control study of pancreatic cancer including patients from Detroit, Atlanta, and New Jersey, established risk factors (cigarette smoking, long-term diabetes mellitus, family history of pancreatic cancer) accounted for 46% of the risk of disease in Black men and 37% in White men, potentially explaining all but 6% of the excess risk among Black patients. Among women, when less accepted risk factors such as moderate/heavy alcohol consumption (>7 drinks per week) and elevated BMI (above the first quartile) were combined with established risk factors, 88% of the risk of disease in Black women and 47% in White women was explained, potentially accounting for all of the excess risk among Black women (38).

## Second-Generation Studies: Explaining Disparities

The studies included in this section identify explanatory variables that might at least in part explain Black-White disparities as outlined below. PSDR investigators and collaborators assessed disparities using different outcome measures such as disparities in receipt of cancer-directed treatment, disparities in stage at diagnosis, disparities in rate of relapse, and disparities in survival including overall and disease-specific survival. The majority of studies utilized multivariable methods in order to determine the contribution of multiple potential confounders on racial disparities in outcomes.

### Disparities in Cancer Treatment

**Table 3** includes studies that evaluate racial disparities in cancer treatment and show mixed results across type of treatment, tumor type and institution. In an analysis of data on treatment for breast cancer from the KCC, no differences or disparities were found related to surgery or radiation, but Black women with regional stage disease were more likely to receive tamoxifen (OR 4.59, 95% CI 1.52-13.9) or chemotherapy (OR 3.10, 95% CI 1.09-8.81), suggesting the presence of other factors related to the need for more aggressive treatment among Black patients. In the same analysis, women with Medicare or Medicaid were more likely to have mastectomy compared to breast conserving surgery, suggesting that older women and women with lower income were less likely to take, or less likely to be offered, the more favored surgical option (39). In another analysis of early stage breast cancer at the HFHS, there were no racial differences in the receipt of adjuvant chemotherapy (OR 1.01, 95% CI 0.72-1.42) or in the timing of receipt of chemotherapy (OR 1.18, 95% CI 0.8-1.74) (40). Lastly, in an analysis of Black-White differences in breast cancer survival at the KCC, there were no racial differences in treatment received or the cost of care, suggesting similar provision of care despite race at a single institution (18).

**Table 3:** Studies focusing on Black-White differences in cancer-directed therapy.

Years of study, Reference	Population	Outcome	Race Groupings	HR, 95% CI	Disparity
1990-6 Banerjee et al. (39)	KCC^1^ N=651	Racial differences in treatment –breast cancer	BCS^2^± RT^3^ *vs.* Mastectomy ± RT White *vs.* Black (ref)	Multivariate Odds Ratios	No racial differences in Breast Conserving surgery *vs.* mastectomy however Black women with regional stage disease more likely to receive tamoxifen or chemotherapy. Multivariate adjustment included race, hormone receptor status, tumor size and grade, positive nodes, age at diagnosis, SES, insurance, marital status and comorbidities.
			Localized	0.81 (0.4-1.65)	
			Regional	1.65 (0.65-4.16)	
			tamoxifen		
			White *vs.* Black (ref)		
			Localized	0.85 (0.35-2.07)	
			Regional	4.59 (1.52-13.9)	
			Chemotherapy White *vs.* Black (ref)		
			Localized	1.3 (0.52-3.26)	
			Regional	3.10 (1.09-8.81)	
1996-2005 Simon et al. (40)	HFHS^4^ N=2234	Racial differences in the use of and timing of adjuvant chemo-therapy- Breast	Chemo	Multivariate Odds Ratio	No Black-White differences in use or timing of adjuvant chemotherapy after adjusting for age, tumor characteristics comorbidities, and SES variables.
			Black	1.01 (0.72-1.42)	
			White	Ref	
			Chemo delay >60 days		
			Black	1.18 (0.8-1.74)	
			White	Ref	
1994-7 Du and Simon (18)	KCC N=588	Racial differences in patterns and costs of care, stage 1-3 - Breast	Surgery (Black *vs.* White):		No Black-White differences in treatment or cost of care at the KCC.
			Lumpectomy	97% *vs.* 96%	
			Lumpectomy + RT	82% *vs.* 76%	
			Mastectomy + RT	25% *vs.* 16%	
			Chemotherapy	41% *vs.* 42%	
			Tamoxifen	71% *vs.* 74%	
			Mean 1-Year total treatment costs (Black *vs.* White)	$16,348 vs.	
				$ 15,120	
2004-14 Beebe-Dimmer et al. (41)	SEER^5^-Medicare N=8828	Treatment pattern by race - *de novo* stage IV prostate cancer; age ≥66yrs	No treatment:	Multivariable Odds Ratios	Black *vs.* White men more likely to have no treatment and more likely to have orchiectomy.
			Black *vs.* White	2.15 (1.7-2.71)	
			Orchiectomy:		
			Black *vs.* White	10.1 *vs.* 6.1%	
1988-1992 Schwartz et al. (27)	DMA^6^-SEER: N=8679	Racial disparities in treatment, OS and DSS - prostate	Localized Black *vs.* White:		Black *vs.* White men less likely to have radical prostatectomy and more likely to have no treatment.
			Radical prostatectomy	16 *vs.* 26%	
			RT	36 *vs.* 38%	
			No treatment	48 *vs.* 36%	
				(p,0.001)	
			Regional Black *vs.* White:		
			Radical prostatectomy	26% *vs.* 47%	
			RT	30% *vs.* 28%	
			No treatment	44% *vs.* 24%	
				(p<0.001)	
2009-10 Xu et al. (42)	SEER-DMA N=260	Treatment choice for localized disease – prostate	Treatment choice: Black *vs.* White:	Multivariable Odds Ratio	Black men were less likely than White men to have surgery or radiation compared to watchful waiting, however there were no differences in choice of surgery or radiation by race.
			Surgery *vs.* WW/AS^7^	0.42 (0.05-0.31)	
			Radiation *vs.* WW/AS	0.13 (0.02-0.69)	
			Radiation *vs.* surgery	0.31 (0.06-1.63)	

^1^KCC, Karmanos Cancer Center.

^2^BCS, Breast conserving surgery.

^3^RT, Radiation therapy.

^4^HFHS, Henry Ford Health System.

^5^SEER, Surveillance, Epidemiology and End Results.

^6^DMA, Detroit Metropolitan Area.

^7^WW/AS, Watchful waiting/active surveillance.

In contrast however, racial disparities in treatment were consistently noted in studies of men with prostate cancer. In three SEER-based collaborative studies, Black compared to White men with prostate cancer were less likely to receive treatment for their cancer. In one study, Black men were less likely to receive any treatment for de-novo stage IV disease (41), or if they received treatment, more likely to have orchiectomy. In another study, Black men were less likely to receive treatment for local or regional stage disease and had worse survival (27). In the third study, Black men were more likely to choose observation only instead of active treatment (42).

In the HFHS study cited earlier, on the influence of co-morbidities on racial differences in outcome for women with endometrial cancer (**Table 2**), Black women were more likely to have no surgery (17% *vs.* 4%, p<0.001), and also more likely to need chemotherapy after surgery (27% *vs.* 20%, p=0.041), again potentially reflecting the more advanced stage and aggressive disease seen in Black women (data not shown in the table) (22). In summary, the studies cited in this section suggest that based on primary site, Black compared to White patients with comparable disease and stage may either be under-treated (less surgical intervention) or receive less desirable treatment (more mastectomy or orchiectomy). Other studies from single institutions show more comparable receipt of treatment across racial groups.

### Disparities in Socioeconomic Status and the Impact on Cancer Outcomes

It is generally acknowledged that there are strong associations between variables that include patient social and economic characteristics, neighborhood characteristics, and cancer outcomes (43). Since individual measures of SES such as income, education and insurance are generally not available in larger population-based studies, PSDR investigators in collaboration with other researchers assessed SES through linkage of geocoded residential addresses to sources of data for larger groups residing in standard geographic regions such as census tract (44) county, or census block, noting the potential for ecological fallacy**** (43, 45).

To account for differences in SES and other neighborhood characteristics, PSDR investigators used the deprivation index, which provides an estimate of the quality of living conditions at census tract levels. The deprivation index is based on the proportion of households without a vehicle; households without a telephone; population over the age of 16 that are unemployed; population living in a residence with more than 1 person per room; and population living below the poverty line (46). A composite index is calculated by adding the value of each of the variables divided by 5 to produce a single index value ranging from 0 to 1, with 0 representing no deprivation and 1 maximal deprivation (17). It should be noted that this method, while providing a broader context for individual SES, is not perfectly valid in that high-deprivation areas can include people of varying economic background (47).

Across a range of studies using data from single institutions or from the SEER registry, Black compared to White patients were more likely to have a primary residence in census tract areas documented as low SES or in areas where a lower proportion of the population had received higher education or had access to medical insurance (15, 17, 19, 40, 48, 49), or to reside in areas with higher measures of deprivation (17, 40, 49). In addition, as reported in one Detroit Metropolitan area-SEER study, Black breast cancer patients were more likely to reside in an area where hospitals provided more care for Medicare and Medicaid patients (13).

In studies using data from the KCC (18), pooled data from case-control studies (50), or from a large US study of postmenopausal women (51), Black compared to White patients had lower levels of educational achievement. In a Detroit Metropolitan Area-SEER analysis of colorectal cancer (CRC), Black compared to White patients were less likely to reside in a census tract area categorized as “professional” (16.5% vs. 42.5%, p<0.001) (15). In a study of women with estrogen and progesterone receptor negative breast cancer at the HFHS, Black compared to White women were ten times more likely to reside in an area with the highest level of deprivation (45.9% vs 4.4%, respectively p< 0.001) (49).

**Table 4** lists studies that include SES as part of a multivariable model. In a Detroit Metropolitan Area -SEER study of racial differences in breast cancer survival, after adjustment for SES (based on census tract level data), and other predictors of survival including socio-demographic and clinical factors, White women with local- and regional-stage disease had better overall survival rates than Black women (p<0.00001); however, for women with distant-stage disease, there were no significant survival differences by race (p=0.3). In this analysis, racial differences in survival were only apparent up to 4 years after diagnosis, but not after 4 years (p=0.6). It is of note that the difference in survival for Black and White women in this analysis was more apparent among women at a younger age at diagnosis with a relative risk (RR) for women < 50 years at diagnosis (RR,1.68, 95% CI 1.27-2.23), that was greater than the RR for women 51 and older at diagnosis (RR, 1.33, 95% CI, 1.13-1.56) (13). In other Detroit Metropolitan Area-SEER studies however, adjustment for SES at the census tract level accounted for all of the racial disparities in overall and disease specific survival for Black and White individuals with CRC (15), and overall survival for women with cervical cancer (48).

**Table 4:** Racial Disparities Studies Which Include Socioeconomic Status as Part of Multivariable Model.

Years of Study, Reference	Population	Outcome	Race Groupings	HR, 95% CI	Disparity
1988-1992, Simon and Severson (13)	DMA^1^ SEER^2^ N=10,502	Overall Survival – Local, Regional and Distant Stage - Breast	Age < 50	Multivariate RR	Blacks had worse overall survival particularly among younger women after adjustment for clinical factors and SES-based on census tract.
			Black	1.68 (1.27-2.23)	
			White	Ref	
			Age 51 +		
			Black	1.33 (1.13-1.56)	
			White	Ref	
1988-1992, Yan et al. (15)	DMA-SEER N-9,078	Overall and disease-specific survival-Colo-rectal	Overall Survival	Multivariate HR	No Black-White differences in overall or disease specific survival after adjustment for clinical and SES-based on census tract.
			Black	1.0 (0.92-1.09)	
			White	Ref	
			Disease specific Survival		
			Black	1.06 (0.94-1.19)	
			White	Ref	
1988-1992 Movva et al. (48)	DMA-SEER N=1,036	Overall Survival for stage I-IV - cervical cancer	Overall Survival	Multivariate HR	No Black-White differences in overall survival after adjustment for clinical factors and SES based on census tract.
			Black	1.12 (0.89-1.42)	
			White	Ref	
1994-1997, Du and Simon (18)	KCC^3^ N=588	Overall Survival and disease free survival -Stage I-III -Breast	Disease Free Survival	Multivariate HR	No Black-White differences in disease free or overall survival after adjustment for clinical factors and co-morbidities (insurance as proxy for SES but not included in the multivariable model)
			Black	1.38 (0.85-2.26)	
			White	Ref	
			Overall Survival		
			Black	1.06 (0.64-1.79)	
			White	Ref	
1996-2005, Roseland et al. (17)	HFHS^4^ N=2,387	Overall Survival – Stage I-III-Breast	Overall Survival	Multivariate HR after adjustment for SES	No Black-White overall survival differences after adjustment for SES based on disparity index
			Black	0.97 (0.8-1.19)	
			White	Ref	
1996-2005, Roseland et al. (49)	HFHS N=542	Overall Survival ER/PR-, Stage I-III-Breast	Overall Survival	Multivariate HR after adjustment for SES	No Black-White overall survival differences only after adjustment for SES based on disparity index. After adjustment for clinical factors and treatment Blacks still had worse outcome.
			Black	1.26 (0.84-1.87)	
			White	Ref	
1988-1992, Schwartz et al. (52)	DMA - SEER N=45,056	Regional + Distant *vs.* Local Stage at diagnosis-Breast, prostate, lung, colorectal and cervical.	Breast CA	Adjusted OR	Black race independently predicted advanced stage after adjustment for SES based on census tract for breast and prostate. No differences for lung, colorectal or cervical
			Black	1.30 (1.17-1.46)	
			White	Ref	
			Prostate CA		
			Black	1.51 (1.35-1.70)	
			White	Ref	
12/2002-1/2013 Lantz et al. (14)	DMA + Los Angeles SEER N=1,700	Stage 0 + I *vs.* Stage II + III - Breast		Adjusted OR	No Black-White differences in stage at diagnosis after adjustment for clinical factors, treatment, diagnostic method and SES-(only study with SES based on individual survey)
			Black	0.79 (0.57-1.1)	
			White	Ref	
2000-2009, Holowatyj et al. (16).	SEER-18 N=28,145	Disease specific survival (age 20-49)-Colon and Rectal	Colon	Adjusted HR	Black-White differences in disease specific survival for young onset colorectal cancer after adjustment for clinical and treatment factors, and SES based on county-level poverty.
			Black	1.35 (1.26-1.45)	
			White	Ref	
			Rectal		
			Black	1.51 (1.37-1.68)	
			White	Ref	
1988-1992, Schwartz et al. (27)	DMA-SEER N=8,679	Influence of co-morbidity and SES on overall and disease specific survival for local and regional stage-prostate	Overall Survival	Adjusted HR	No overall survival differences for Black and White men with local or regional stage disease, however disease specific survival differences for men with local stage, after adjustment for treatment and SES-based on census tract.
			**Local**		
			Black	1.03 (0.96-1.11)	
			White	Ref	
			**Regional**		
			Black	0.90 (0.73-1.13)	
			White	Ref	
			Disease Specific		
			**Local**		
			Black	1.35 (1.17-1.65)	
			White	Ref	
			**Regional**		
			Black	0.83 (0.57-1.20)	
			White	Ref	
2002-2007 Schwartz et al. (34)	DMA-SEER: N=951	Overall survival -r renal cell carcinoma	OS:	Adjusted HR	No survival differences after adjustment for clinical, treatment factors, co-morbidities and SES based on deprivation index.
			Black	0.93 (0.65-1.35)	
			White	Ref	
			**<65y at diagnosis**		
			Black	1.14 (0.71-1.85)	
			White	Ref	
			**Tumor ≤4cm:**		
			Black	1.15 (0.67-1.98)	
			White	Ref	

^1^DMA, Detroit Metropolitan Area.

^2^SEER, Surveillance, Epidemiology and End Results.

^3^KCC, Karmanos Cancer Center.

^4^HFHS, Henry Ford Health System.

Similarly, results from single-institution studies showed no racial differences in survival after accounting for SES. These included a KCC study which showed no Black-White differences in breast cancer disease-free survival (HR 1.38, 95% CI 0.85-2.26) or overall survival (HR 1.06, 95% CI 0.64-1.75) (18), and in the HFHS analysis that showed no breast cancer survival differences after adjustment for neighborhood deprivation index (HR 0.97, 95% CI 0.8-1.9) (17). In a sub-analysis of Black and White women with estrogen and progesterone receptor negative breast cancer at the HFHS, there was also no overall survival disparity after adjustment for deprivation index (HR 1.26, 95% CI 0.84-1.87) (49).

However, other studies showed racial disparities even after controlling for economic and social factors. In a Detroit Metropolitan Area-SEER study after adjusting for SES based on census block, Black patients still had more advanced stage at diagnosis (regional or distant *vs.* local) among women with breast cancer (OR 1.30, 95% CI 1.17-1.46) and men with prostate cancer (OR 1.51, 95% CI 1.35-1.70) (52). However, differences in stage at presentation were not seen in an analysis of women with stage 0 to III breast cancer using the Detroit and Los Angeles SEER registries where after adjusting for SES based on individual questionnaire, and method of detection, there were no significant differences between Black and White women in diagnosis of stage 0+I disease *vs.* II+III (14).

In a SEER-18 site study of early onset CRC, after adjusting for SES based on the proportion of individuals below 200% of the poverty level at the county level as well as clinical and treatment factors, Black compared to White patients continued to have worse disease specific survival for both colon (HR 1.35, 95% CI 1.26-1.45) and rectal cancers (HR 1.51, 95% CI 1.37-1.68) (16). A Detroit Metropolitan Area–SEER prostate cancer study showed that after adjustment for SES based on census block data, there were no significant differences for Black and White men for overall survival; however, Black men with localized disease continued to have worse disease specific survival (HR 1.35, 95% CI 1.17-1.65) (27). Lastly, in a Detroit Metropolitan Area-SEER case-control study of RCC that adjusted for SES based on deprivation index and also adjusted for clinical factors and co-morbidity, there were no racial differences in overall survival (HR 0.93, 95% CI 0.65-1.35)**** (34).

Other studies that did not take into account SES showed varying results (not shown in Table). In a KCC analysis of men who had radical prostatectomy for clinically localized disease, after multivariable adjustment, Black men continued to have worse progression-free survival than White men (HR 2.35, 95% CI 1.63-3.4), p<0.0001 (53). Another study comparing the pre- to post-prostate screening era (PSA testing), another “natural experiment” over time, showed that Black men diagnosed in the pre-PSA era (1973–1994) had higher mortality than White men for all age groups; however this difference in survival disappeared in the post-PSA era (26).

In summary, PSDR investigators in collaboration with others have conducted a wide range of studies using data from either single centers in Detroit or across multiple sites using SEER or other larger data bases to better understand and explain racial health disparities. In general, the studies cited demonstrate that Black compared to White patients with cancer present with more advanced and biologically aggressive disease, are more likely to live in economically and socially deprived areas and are more likely to be diagnosed with co-morbid medical conditions which has the potential to hamper accessibility to optimal cancer-directed treatment. It is also possible that Black patients with co-morbid medical conditions are less likely to be offered treatment as a White person with the same co-morbidity.

While a number of studies have sought to evaluate factors such as differences in treatment or SES that might explain some of the disparities in cancer outcomes noted, these types of first- and second-generation disparities studies are largely descriptive and serve only to document the extent of the problem or identify potential factors underlying the disparities. Further, while controlling for SES is an accepted practice in studies of treatment disparities, two important caveats should be taken into consideration. First of all, SES disparities can be easily traced to legacies of institutional and structural racism and therefore controlling for SES is an attempt to control for one of the consequences of racism which remains unresolved. Secondly, in the process of controlling for disparities, researchers in a sense are creating a world where Black and White people have the same SES, which does not reflect reality.

## Patient-Physician Communication and Disparities: First, Second and Third Generation Studies

In order to identify potential ways to address and intervene to reduce racial disparities, investigators in the PSDR in collaboration with other researchers, have conducted studies evaluating differences in clinical communication during patient-physician interactions with Black compared to White patients. In this section, we will present first-generation evidence of disparities in patient-physician communication and then second-generation studies looking at potential factors explaining these disparities and the impact of disparities on cancer outcomes. We will then describe third-generational interventional studies designed to mitigate these disparities in communication.

### First-Generation Communication Studies

Clinical communication involving patients, their companions, and their providers plays a critical role in patient-centered care and outcomes (54, 55). Racial disparities in patient-physician communication are well-documented and have been associated with racial disparities in cancer treatment and mortality (56, 57). In research that used video-recordings of patient-physician treatment discussions (after written consent) (58), PSDR researchers have investigated disparities in clinical communication during interactions between non-Black oncologists and their Black patients (59–62). They have also studied the influence of race-based attitudes (e.g., oncologist implicit racial bias, patient suspicion of medical care) on those interactions (61, 62).

As part of this research, PSDR researchers conducted a mixed-methods analysis of 109 video recordings to investigate patient and companion question-asking during interactions with oncologists. Findings showed that compared to White patients, Black patients asked fewer total questions, fewer direct questions, and were less likely to have a companion with them to contribute to question-asking and information-exchange. Findings from this research suggest that these differences in question asking may diminish the quality of information exchange during interactions with Black patients and that Black patients may receive less information from their oncologists (63).

A more recent study of video-recorded interactions between 114 Black patients and non-Black oncologists found that having a companion(s) during the interaction had a positive impact on patient-oncologist interactions. These included oncologists spending more time with the patient and using more patient-centered communication with patients who brought a companion (64). Given documented racial disparities in communication, the presence of companions may be especially beneficial to Black patients. Findings from these studies suggest that provider-level and system-level interventions may be used to encourage patients to participate actively in clinical interactions and to bring supportive companions to assist them in exchanging information with physicians. For example, oncologists could be trained to elicit patient questions and answer them directly and compassionately, and hospitals could encourage companion participation by facilitating video conferencing with companions who may not be able to attend visits.

Another study used linguistic discourse analysis to better understand communication about clinical trials in video-recorded interactions with Black and White patients and their medical oncologists. Findings showed that interactions with Black patients were shorter; the topic of clinical trials was less frequently mentioned; and, when clinical trials were mentioned, less time was spent discussing them (65). Differences were also observed in the discussion of some aspects of consent to clinical trials. Specifically, oncologists and Black patients spent less time discussing the purpose of the trial, risks and benefits, and alternatives to participating in the trial; however, they spent more time discussing the voluntary nature of trials (65). These findings are particularly problematic if this type of communication about clinical trials is the norm for Black patients with cancer (66) because it suggests that Black patients are not receiving adequate information to make an informed decision about participating in a clinical trial. These and similar findings led to intervention studies, described below, to improve the quality of communication during interactions in which clinical trials may be discussed.

### Second-Generation Communication Research

Several investigations have focused on the influence of patient and physician race-based attitudes. With regard to physician race-based attitudes, PSDR investigators showed that oncologists with higher implicit bias (i.e. favoring White people) were more likely to have shorter interactions with Black patients than oncologists with lower implicit bias. Further, their communication was perceived by patients as less patient-centered and by patients and independent observers as less supportive with their Black patients. This in turn led to less patient confidence in treatment recommendations and greater perceived difficulty in completing treatment (61). With regard to patient race-based attitudes, using the Group-Based Medical Mistrust scale developed by a PSDR investigator (67), relationships were found between Black patients’ group-based medical suspicion (67) and their attitudes about adherence and decisional control (62). Other work found that high levels of group-based medical suspicion among Black patients was associated with more negative evaluations of physicians and recommended treatment (68). Patient mistrust of medical care and lack of trust in physicians were also associated with not only how much Black patients spoke during medical interactions but also the valence of the words they used. High levels of patient mistrust was also associated with less favorable physician perceptions of Black patients, which, in turn, affected physician perceptions of how well these patients would tolerate treatments (68). A more recent study found that Black men with prostate cancer had higher levels of group-based medical suspicion than White men, and this race-based attitude was associated with less willingness to discuss clinical trials with their physicians (69). More current research is examining how patient and oncologist nonverbal communication may be associated with these race-based attitudes (70, 71).

### Third-Generation Communication Interventions

In an attempt to mitigate these disparities in communication and the influence of race-based attitudes, PSDR investigators have developed and tested communication interventions. One type of low-cost and effective intervention tested has been question prompt lists (QPL). QPLs are simple communication tools designed to promote active participation in clinical interactions. QPLs are provided to patients before a clinical interaction and include a list of questions patients can consider asking their physician in a specific clinical context (72–75). Using a community engagement process to aid in its development, PSDR investigators created and tested a QPL for patients considering chemotherapy (76, 77). In a trial with 114 Black patients randomized to receive standard of care, a QPL brochure, or a QPL brochure and the assistance of a coach, video recordings of patient-physician interactions and post-interaction, patient surveys demonstrated that the QPL was feasible and acceptable. The QPL also increased observer coded patient active participation and information exchange in treatment discussions with their medical oncologist (78). In ongoing studies, researchers are currently testing the effectiveness of a QPL designed to improve clinical trials discussions along with a companion intervention for oncologists (78), and an app-based QPL focused on cancer treatment costs (79).

## Towards Fourth Generation Disparities Research

The current paper has framed KCI research in the context of first-, second,- and third -generation disparities research. We are now laying the foundation for fourth-generation research, which is rooted in justice and action to eliminate disparities. Fourth generation research may be guided by public health critical race praxis (PHCR): “…a semi-structured process for conducting research that remains attentive to issues of both racial equity and methodologic rigor” (80). At the core of PHCR is race consciousness, which requires attention to racial dynamics within the research context as well as the outer world, and the role of racism in cancer health inequity (80).

Thomas et al. (12) assert: “In fourth-generation research, guided by PHCR, it is essential to remember that the goal is ultimately to take action to eliminate health disparities. In that context, the voice of community members is an absolute necessity. Fourth-generation research is deeply rooted in community and the racialized context of the populations who reside within them.” The need for community voice in research guides KCI’s Office of Cancer Health Equity and Community Engagement and its Michigan Cancer HealthLink program, which engages diverse populations throughout the state to build public interest, involvement, and community capacity to collaborate in cancer-related research. Michigan Cancer HealthLink is an academic-community partnership that uses a participatory research approach to facilitate collaboration between community members and researchers through an iterative process of problem definition, problem solving, and evaluation. Partnership activities also focus on skill development, resource mobilization, and relationship building. HealthLink is based on a network of Cancer Action Councils or CACs: groups of cancer survivors, caregivers, and advocates who use their local knowledge and expertise to reduce the burden of cancer in underserved communities. There are currently 9 CACs with over 100 members in six cities across four counties in Michigan, representing Black American, Arab American, young survivor, and LGBT+ communities (81).

Through Michigan Cancer HealthLink, we are setting the stage for fourth generation research to eliminate the disparities that disadvantage Black Americans. These efforts are informed by the principle of voice: the privileging of marginalized persons’ contributions to discourses (81). The CACs are a key way to amplify the voices of Black Americans in our work. Using semi-structured methods, CACs identify and develop research priorities that they feel are most relevant to their specific communities. CACs also receive training in research methods to prepare them to actively partner with our researchers and contribute to development of research ideas, design, and implementation. In short, HealthLink is an infrastructure that supports action-oriented disparities research by building research capacity in Black American communities and soliciting different perspectives that can challenge, supplement, and even replace the traditional, academic perspectives that tend to dominate disparities work. As Ford et al. (80) assert, “[Voice] helps to illuminate disciplinary blind spots that are otherwise imperceptible from within a discipline’s mainstream. It increases understandings of minorities’ lived experiences, which improves operationalization of constructs, development of effective interventions and creation of an equitable society.” As we apply a PHCR approach, we increasingly distance ourselves from the perspective that Black American communities are mainly environments that drive racial disparities in cancer risk, care, and outcomes. When viewed through the PHCR lens, these communities are vital hubs for resources, opportunities, partners, and solutions to achieve equity.

## Discussion

Researchers in KCI’s PSDR, along with their collaborators, have spent the past three decades investigating racial disparities in cancer incidence, treatment, and outcomes among Black and White patients in Southeast Michigan, with a specific focus on the Detroit area, a city with a majority Black population. The studies document the pattern of more aggressive disease seen in Black compared to White cancer patients, as well as higher rates of co-morbid medical conditions and differences in treatment and communication, which have additional adverse effects on cancer outcomes. These findings suggest that in order to reduce or eliminate racial disparities in cancer outcomes, it is also of utmost importance to address larger questions of inequality inherent in the legacy of structural racism in the US, along with disparities across the spectrum of chronic comorbid and medical conditions, which have an disproportionately negative impact on Black people (4).

To date, abundant research on cancer disparities and inequities in health outcomes has been published, as exemplified by the recent review by Zevala et al. (82). This review includes a detailed description of disparities in cancer incidence, mortality, health care screening, treatment and tumor biology experienced by almost every racial and ethnic minority group in the United States, and across both common and uncommon cancers. Consistent with the research reported here, Zevala et al. provided explicit information on potential causative factors and steps needed to mitigate racial and ethnic disparities. Given the demographics of Detroit, our review focused on studies inclusive of Black and White cancer survivors outlining disparities similar to that experienced by other groups (82). Future work in the Detroit area should focus on structural racism and disparities in cancer care experienced by additional marginalized and minority groups in the region including the large Mexican and Arab American populations. One prime example of structural racism is the status of residential housing in the US (4, 83). Black cancer patients are more likely to live in geographic areas with lower levels of aggregate SES, and consequently are faced with less access to high-quality medical care, resulting in lower quality of care. While structural racism may be difficult to address in the short term, providing transportation and easier access to care may be a first step with longer-term goals of equitable housing and insurance coverage. Recent efforts on the part of academic health centers including WSU and the KCI to better understand the underlying influence of social determinants on health care outcomes can provide new structures and options in which to train health care professionals to provide more equitable health care for all racial and ethnic groups (84, 85).

Other underlying reasons for Black-White health disparities include the legacy of fewer and lower quality medical services being available in the areas where many Black people live (5–7, 86); poor employment opportunities; and inadequate and unhealthy housing and inadequate or substandard education opportunities that have historically disadvantaged the Black community and have both directly and indirectly contributed to poor health care outcomes (86). Similarly, a historic loss of population for socioeconomic reasons or excess mortality from chronic health conditions has disproportionately burdened the Black population over time (87). Our research has demonstrated a few examples of “natural experiments” documenting the widening gaps in survival and treatment options for Black and White women with breast cancer over several birth cohorts (29), and a diminution in the prostate cancer mortality gap marked by the institution of PSA screening (26). Future work in Detroit should take advantage of other natural experiments such as new health care policies such as the Affordable Care Act, or other interventions that may shed light on how structural factors impact racial and ethnic differences in outcomes. In order to reduce Black-White disparities in cancer outcomes, we propose multi-level interventions such as community engagement, recruiting more diverse clinical staff (88), and conducting system-wide anti-racism training (85). Our research points to the benefit of interventions that are relatively easy to implement and could have an immediate impact on the quality of and better access to care, including Question Prompt lists, brief language-appropriate educational videos and the inclusion of Lay Health Advisors (89) and patient navigators as essential components of the health delivery system. Such efforts would also be easily adaptable to additional underserved populations.

At the KCI, the Detroit Research on Cancer Survivorship (ROCS) study is the only National Cancer Institute-funded survivorship cohort of Black cancer survivors in the US (90). Studies of this cohort (enrollment goal 5,000) are seeking to elucidate the specific influence of systematic and structural racism at the community and individual levels on the emotional and physical health, health and screening behaviors, and quality of life of Black cancer survivors (91–93) An understanding of race-related factors underlying and maintaining health-disparate outcomes is essential for developing interventions and initiatives that could reduce current inequalities in diagnosis, treatment, and survival, and improve the quality of cancer survivorship in Black men and women with cancer. Knowledge gained from research on racial health disparities among cancer patients and those with other medical conditions can also help to eradicate disparities in additional groups in the future.

## Author Contributions

SR: She conducted the pub med review of all of the articles included in the review, constructed the tables and helped edit and write the manuscript. LH: She wrote and organized the section on physician communication. She also was responsible for extensive editing. LP: Extensive editing with a focus on racial disparities and the physician communications section. KS: Extensive editing with a focus on the epidemiologic studies. FH: Extensive editing with a focus on the physician communications section. HT: She wrote the section on fourth generation research and edited the rest of the manuscript. JB: Editing with a focus on area based measures of SES. MC: Editing. AS: Editing and advise as to formatting and structure. SE: Extensive editing. Focused on the physician communication section. All authors contributed to the article and approved the submitted version.

## Conflict of Interest

The authors declare that the research was conducted in the absence of any commercial or financial relationships that could be construed as a potential conflict of interest.
